# Teaching and learning strategies for the derivative in engineering education: A scoping review protocol

**DOI:** 10.1371/journal.pone.0360253

**Published:** 2026-10-22

**Authors:** Maritza Katherine Galindo Illanes, Denise Deyanira Chamorro Manríquez, Cristian Herrera Valdés

**Affiliations:** 1 Universidad San Sebastián, Chile; 2 Universidad Católica de la Santísima Concepción, Chile; 3 Universidad San Sebastián, Chile; Federal University of Ceara, BRAZIL

## Abstract

The derivative is a fundamental concept in engineering education, as it enables students to model and interpret phenomena related to rates of change, optimization, and the analysis of physical processes. However, its teaching in higher education often emphasizes decontextualized algebraic procedures, with limited integration of authentic engineering problems and interactive digital technologies. This protocol outlines a scoping review aimed at mapping the available evidence on teaching and learning strategies for the derivative in undergraduate engineering education that incorporate authentic engineering problems, with or without the support of digital technologies. The review will follow the methodological framework proposed by the JBI Manual for Evidence Synthesis and the reporting guidelines of PRISMA-ScR. The review is guided by the PCC framework: (P) undergraduate engineering students; (C) didactic strategies for teaching and learning the derivative grounded in authentic engineering problems, with or without the support of digital technologies; and (C) higher education engineering programs. Searches will be conducted in Scopus, Web of Science, ERIC, and SciELO for peer-reviewed publications published between 2000 and 2026 in English, Spanish, and Portuguese. Two reviewers will independently screen and select studies using Rayyan, and disagreements will be resolved by consensus. Data will be extracted using a pilot-tested data extraction form and synthesized through descriptive statistics, thematic categorization, and narrative synthesis. The review will provide a comprehensive mapping of didactic strategies, types of authentic problems, and reported learning difficulties related to the derivative in engineering education. The findings are expected to inform instructional design and support the development of evidence-based teaching practices in calculus courses for engineering students.

## 1. Introduction

The derivative plays a fundamental role in engineering education, providing a key mathematical tool for modeling rates of change, analyzing physical phenomena, and solving optimization problems [1]. Its application through authentic engineering problems has been recognized as an effective way to strengthen the connection between calculus instruction and engineering practice [2]. This mathematical concept connects mathematical reasoning with authentic engineering problems, where understanding it is essential for interpreting technical phenomena and making decisions based on quantitative models [3]. The ability to apply the derivative in real contexts enables future engineers to design efficient and sustainable solutions, thereby improving the quality and effectiveness of engineering projects.

Despite its central role in engineering education, several studies [1,3,4] have documented persistent difficulties in teaching and learning the derivative in higher education. In particular, the predominance of operational conceptions and the use of symbolic procedures have been observed, together with limited integration of graphical, algebraic, and verbal representations within instructional tasks and explanations [1,4,5]. There is also evidence of limited understanding of the limit concept as the foundation of the derivative, as well as difficulties with covariational reasoning required to interpret the coordinated variation of quantities in dynamic situations [3,6,7]. These difficulties hinder the transfer of knowledge to authentic or plausible engineering situations, such as resource optimization and the interpretation of dynamic phenomena.

To address these challenges, recent studies have proposed didactic strategies that promote conceptual understanding of the derivative [8,9]. Among the most prominent approaches are the use of interactive digital technologies —such as GeoGebra, process simulators, and graphic visualization tools [10,11]— and the incorporation of authentic engineering problems that support the representation, exploration, and integration of multiple meanings of the derivative as a mathematical object. Digital technologies have been shown to support the visualization, manipulation, and dynamic exploration of mathematical phenomena within contextualized learning environments. Similarly, authentic problems situated in real or plausible professional contexts support the interpretation and solution of complex engineering situations. For example, the literature describes tasks focused on resource optimization (cost-benefit), the analysis of variations in water flow, and the interpretation of rates of change in physical processes using realistic data drawn from engineering contexts [5,10]. In engineering education, differential calculus courses are implemented across heterogeneous curricular contexts, including variations in program duration, course sequencing, competency-based approaches, and instructional modalities [12,13]. This diversity reinforces the need to map how the derivative is taught and learned across different undergraduate programs and disciplinary contexts.

Furthermore, a substantial body of evidence from engineering education supports the use of active learning approaches grounded in authentic and contextualized problem-solving, including problem-based learning, project-based learning, flipped classrooms, and interactive simulations. These approaches have been associated with improved academic performance and significant reductions in failure rates compared with traditional lecture-based instruction [14–18]. However, important gaps remain regarding how these approaches have been implemented specifically for teaching the derivative, the types of authentic engineering problems used in calculus instruction, and the role that digital technologies play in these instructional approaches.

Despite these advances, the evidence on teaching and learning strategies for the derivative in engineering education that integrate authentic engineering problems, with or without digital technologies remains fragmented and heterogeneous. As a result, it is difficult to develop a comprehensive understanding of the current state of the evidence and to identify research trends, knowledge gaps, and opportunities for instructional design and instructional practice. Accordingly, conducting a scoping review was considered appropriate to map the available literature, characterize its main features, and clarify concepts and practices across diverse educational contexts. Scoping reviews are designed to map the scope, nature, and characteristics of the available evidence without critically appraising the methodological quality of the included studies [19–21].

A preliminary examination of review studies identified during the development of this protocol, complemented by exploratory searches of review literature indexed in Scopus and Web of Science during manuscript revision using combinations of the terms “derivative,” “differential calculus,” “engineering education,” “teaching,” “learning,” and “review,” suggests that the specific intersection addressed by the present scoping review—namely, didactic strategies for teaching the derivative through authentic engineering problems in undergraduate engineering education—has not been systematically synthesized in the existing review literature.

Several reviews have examined active learning across science, technology, engineering, and mathematics (STEM) higher education. Freeman et al. [15] conducted a meta-analysis demonstrating that active learning increases student performance across STEM disciplines; however, their review was not restricted to engineering programs, did not focus on a specific mathematical concept, and did not examine authentic engineering problems as a distinct instructional approach. Similarly, Prince [16] reviewed active learning in engineering education and discussed general pedagogical approaches such as cooperative learning, problem-based learning, and project-based learning, without addressing the teaching of differential calculus or the derivative specifically.

Reviews focused on technology-enhanced learning have likewise remained at a broader level. Reis et al. [22] synthesized evidence regarding immersive technologies in engineering education, while Sailer et al. [23] conducted a second-order meta-analysis of learning activities in technology-enhanced higher education. Although both reviews provide valuable insights into educational innovation, neither focuses on the derivative as a mathematical object nor on authentic engineering problems as a pedagogical context for calculus instruction. Similarly, Sukackė et al. [24] systematically reviewed active learning approaches in engineering education, including problem-based, project-based, and challenge-based learning, without focusing specifically on the derivative.

Within mathematics education, research concerning the derivative has mainly focused on conceptual understanding, students’ difficulties, representations, and instructional processes [1,3–5,10,25,26]. To the best of our knowledge, no review was identified that simultaneously addressed (a) the derivative as the focal mathematical concept, (b) authentic engineering problems as the instructional context, and (c) undergraduate engineering students as the target population.

Consequently, the present scoping review addresses a unique combination of three dimensions: the mathematical object (the derivative), the instructional vehicle (authentic engineering problems, with or without digital technologies), and the educational population (undergraduate engineering students). Mapping this evidence is a necessary first step toward understanding the current state of knowledge, identifying research gaps, and informing future instructional design and research in engineering mathematics education.

The aim of this study is to map the available evidence on didactic strategies for teaching and learning the derivative in engineering education through authentic engineering problems and to identify the types of authentic engineering problems used and the main difficulties reported in students’ understanding and application of the derivative.

**Research question:** What evidence does the academic literature provide on didactic strategies that use authentic engineering problems, with or without the support of digital technologies, for teaching and learning the derivative among engineering students in higher education?

To address the study objective more precisely, the following sub-research questions are proposed:

What types of authentic engineering problems are used in the teaching and learning of the derivative in higher education engineering programs?

What didactic strategies have been implemented to teach the derivative through authentic problems in engineering education?

What difficulties are reported in students’ understanding and application of the derivative in these contexts?

What role do digital technologies play in didactic strategies based on authentic problems for teaching the derivative?

## 2. Methodology

The review will follow a systematic sequence of stages: (i) formulation of the research question and eligibility criteria using the Population–Concept–Context (PCC) framework; (ii) development and implementation of the search strategy; (iii) reference management and deduplication; (iv) independent screening of studies by two reviewers; (v) systematic data extraction (charting); and (vi) narrative and thematic synthesis of the evidence. Each stage will be documented using the corresponding instruments, including the data-charting form, the PRISMA-ScR flow diagram, and the completed PRISMA-ScR checklist.

### 2.1 PCC framework

The review is guided by the Population–Concept–Context (PCC) framework, which is recommended for scoping reviews.

- Population (P): undergraduate students enrolled in engineering programs in higher education.
- Concept (C): didactic strategies for teaching and learning the derivative or differential calculus based on authentic engineering problems, with or without digital technologies.
- Context (C): higher education engineering programs.

This framework will guide the development of the eligibility criteria and the search, study selection, and analysis processes throughout the review.

### 2.2 Eligibility criteria

Studies will be included if they involve undergraduate students enrolled in engineering programs and focus on teaching and learning the derivative or differential calculus in higher education through at least one authentic engineering problem, as operationally defined below by criteria A–D. The presence of an authentic engineering problem is therefore a mandatory inclusion criterion, whereas the use of digital technologies is optional and will be treated as a characteristic of the didactic strategy rather than as an eligibility requirement. Eligible sources will include peer-reviewed journal articles, academic book chapters, and full papers published in peer-reviewed conference proceedings. Eligible study types will include empirical studies, instructional experiences, intervention proposals, theoretical studies, and review studies, provided that they meet the Population, Concept, and Context eligibility criteria described above.

The review will include studies published between 2000 and 2026, a period characterized by sustained transformation in higher education and, particularly, in engineering education. These decades have witnessed the incorporation of active learning approaches, digital technologies for learning, competency-based instructional design, and an increasing emphasis on flexible and innovative education [27,28]. These developments have fostered teaching approaches that reconsider the role of differential calculus and the derivative in engineering education while promoting learning experiences more closely connected to professional practice.

Works published in English, Spanish, and Portuguese will be considered, as these languages account for a substantial proportion of the published research in mathematics education and engineering education in Europe and Latin America.

### Definition of didactic strategy

For the purposes of this review, a didactic strategy refers to any planned instructional approach, pedagogical intervention, teaching sequence, learning activity, digital technology, or instructional design intentionally used to support students’ understanding and application of the derivative. Examples may include problem-based learning, project-based learning, inquiry-oriented activities, modeling tasks, technology-enhanced instruction, simulations, visualization tools, or instructional sequences specifically designed for differential calculus. The didactic strategy constitutes the pedagogical approach adopted by the study, whereas authentic engineering problems represent the contextualized tasks through which the strategy may be implemented.

### Definition and operationalization of authentic engineering problems

For the purposes of this review, authentic engineering problems are conceptualized as tasks situated in realistic engineering contexts that require students to engage with mathematical ideas in ways that resemble professional engineering practice. Authenticity is understood not merely as the presence of a real-world context [29], but as the meaningful integration of disciplinary knowledge, decision-making, and interpretation within an engineering situation [30,31]. To ensure consistent identification of authentic engineering problems during study selection and data extraction, the following operational definition was developed for this review. A task will be classified as an authentic engineering problem when it simultaneously satisfies the following four criteria:

A. Mathematical centrality. The derivative—understood as a function, a rate of change, a linear approximation, or a tool for optimization—is the primary mathematical object that the task requires students to calculate, construct, interpret, or apply. Tasks in which the derivative appears only incidentally, as a by-product of another procedure, or is provided as a given result without student engagement in its use will not satisfy this criterion.
B. Engineering situatedness. The context of the task corresponds to a phenomenon, system, process, or decision recognizable as belonging to at least one engineering discipline (e.g., civil, mechanical, electrical, chemical, industrial, or systems engineering). This criterion is satisfied when the engineering context is necessary for understanding the task and cannot be replaced by an abstract mathematical setting without altering its meaning.
C. Empirical grounding of data. The numerical values used in the task are drawn from real measurements, technical standards, empirical records, or are explicitly described as representative of plausible engineering conditions. Tasks based on arbitrary numerical values whose representativeness is neither stated nor reasonably inferable from the disciplinary context will not satisfy this criterion.
D. Functional use of the derivative. The application of the derivative produces a result that is meaningful within the engineering context described, such as identifying an optimal design parameter, quantifying the rate of a physical process, predicting system behavior, or supporting a technical decision. Tasks in which the derivative is used solely to practice differentiation procedures without interpretation of the result within the engineering situation will not satisfy this criterion.

A study will be considered to contain authentic engineering problems when it reports at least one task satisfying criteria A–D simultaneously. Studies that refer to authentic, contextualized, or real-world problems only in general terms, without providing sufficient information to assess these criteria, will not be classified as involving authentic engineering problems.

### Exclusion criteria specific to authenticity

A task or study will be excluded on the basis of insufficient authenticity when any of the following conditions apply:

(E1) The engineering context is decorative or superficial, meaning that it can be removed without affecting the mathematical structure or solution process.

(E2) The task belongs to a general mathematics or physics context without an explicit connection to engineering practice or engineering education.

(E3) The derivative is provided as a given result rather than being constructed, interpreted, or applied by students.

(E4) The study does not provide a sufficiently detailed description of the task to allow assessment of criteria A–D.

When a study reports multiple tasks and only some satisfy criteria A–D, the study will remain eligible provided that the authentic tasks form part of the instructional strategy under investigation and are described in sufficient detail to allow data extraction and analysis.

### Reviewer decision procedure

To support consistent application of the eligibility criteria, two reviewers will independently assess the titles, abstracts, and full texts of candidate studies. During title and abstract screening, records will be retained whenever eligibility cannot be determined with certainty based on the available information. Full-text records will then be assessed against the eligibility criteria using a standardized screening form.

Before the formal screening process, the reviewers will conduct a pilot assessment of an initial sample of records to ensure a consistent interpretation of the eligibility criteria and to refine the screening process where necessary.

Disagreements regarding study eligibility or the classification of tasks as authentic engineering problems will be resolved through discussion and consensus. If consensus cannot be reached, a third reviewer will be consulted. Reasons for exclusion at the full-text stage will be documented and reported in the PRISMA-ScR flow diagram. Reviewer decisions and discussions will be recorded to ensure transparency and consistency throughout the screening process.

Grey literature sources will not be included (e.g., unpublished theses, technical reports, institutional documents, preprints, or conference abstracts without an associated full peer-reviewed paper). Although scoping reviews may include grey literature, this review is restricted to the eligible academic sources defined above: peer-reviewed journal articles, academic book chapters, and full papers published in peer-reviewed conference proceedings. This decision aims to ensure transparency, reproducibility, and comparability in the review process and is explicitly described to maintain methodological traceability [19,32].

### 2.3 Search strategy

The search will be conducted in Scopus, Web of Science (WoS), ERIC, and SciELO, which were selected for their relevance to higher education, engineering education, and mathematics education research. Following the JBI Manual for Evidence Synthesis [32], and reported in accordance with the PRISMA-ScR [19], the search strategy was developed using the Population–Concept–Context (PCC) framework. It combines free-text terms and controlled vocabulary organized into three conceptual domains corresponding to the target population (engineering education), the central concept (the derivative and differential calculus), and the educational context (higher education). The main search terms for each PCC domain are summarized below, while the complete reproducible search strategies, including database-specific syntax, controlled vocabulary where applicable, and additional synonyms, are provided in Annex 1 (Reproducible search strategy – JBI spreadsheet). These domains were combined using Boolean operators to maximize the retrieval of potentially relevant studies while maintaining conceptual coherence across databases.

1. Population: “engineering students*”, “engineering education”, “undergraduate engineering”, “engineering program*”.
2. Concept: derivat*, “differential calculus”, “rate of change”, “tangent line”, “optimization problem*”, “authentic problem*”, “real-world problem*”, “teaching strateg*”, “didactic strateg*”, “instructional strateg*”, “teaching method*”, “instructional design”, “pedagogical approach*”, “teaching practice*”, “learning activit*”, “learning task*”.
3. Context: “higher education”, universit*, college*, undergraduate*, postsecondary.

An example of the search string used in Web of Science (WoS) is presented below:

TS = ((“engineering student*” OR “engineering education” OR “undergraduate engineering” OR “engineering program*”) AND (derivat* OR “differential calculus” OR “rate of change” OR “tangent line” OR “optimization problem*” OR “calculus education” OR “teaching strateg*” OR “didactic strateg*” OR “instructional strateg*” OR “teaching method*” OR “instructional design” OR “pedagogical approach*” OR “teaching practice*” OR “learning activit*” OR “learning task*” OR “authentic problem*” OR “real-world problem*” OR “real-life problem*” OR “authentic task*” OR “applied problem*” OR “contextualized problem*” OR “engineering context*” OR “simulation context*”) AND (“higher education” OR universit* OR college* OR undergraduate* OR postsecondary)).

This strategy will then be adapted to the syntax, search fields, and indexing systems of each database. In the case of ERIC, its controlled vocabulary (ERIC Thesaurus) will be used, whereas databases such as Scopus and Web of Science will rely on their respective search fields and subject categories. In all cases, the same conceptual structure and semantic groups described above will be preserved.

In addition, the snowballing technique will be applied to complement the database search. This technique will include: (i) screening the reference lists of the included studies (backward snowballing), and (ii) identifying studies citing the included studies (forward snowballing), using Scopus and Google Scholar.

The combined use of database searching, database-specific adaptations, and snowballing aims to ensure a comprehensive, transparent, and reproducible identification of the available evidence while minimizing the risk of omitting relevant studies in this interdisciplinary field, where terminology, indexing practices, and database coverage may vary.

### 2.4 Study selection and management

References identified through the database searches will be imported into the Rayyan QCRI platform [33] to manage the study selection process, including duplicate detection and independent screening by reviewers according to the predefined eligibility criteria.

The selection process will be conducted in two phases:

- Title and abstract screening, performed independently by two reviewers with experience in mathematics education and scoping review methodology, based on the predefined eligibility criteria.
- Full-text assessment, applying the established criteria.

Before formal screening begins, the reviewers will conduct a pilot assessment of an initial sample of records to ensure a consistent interpretation of the eligibility criteria and refine the screening process if necessary.

Once the independent screening has been completed, Rayyan will identify conflicts or discrepancies, which will be resolved through discussion and consensus between the reviewers.

If such disagreements persist, a third reviewer will be consulted. This procedure promotes consistency, transparency, and reproducibility throughout the study selection process, in accordance with the recommendations of the JBI Manual for Evidence Synthesis [32].

During the full-text assessment stage, the reasons for excluding studies that do not meet the eligibility criteria will be documented and reported.

These decisions will be systematically documented and presented in the PRISMA-ScR flow diagram [19], which will report the number of records identified, duplicates removed, records excluded during title and abstract screening, and full-text records assessed for eligibility, including the reasons for exclusion at this stage.

### 2.5 Data extraction

Data will be extracted independently by two reviewers using a data extraction form developed by the research team to ensure consistency, transparency, and traceability throughout the review process.

Before formal data extraction begins, the reviewers will pilot the data extraction form using a small sample of included studies. This pilot exercise will allow the reviewers to assess the clarity and completeness of the extraction categories and to refine the data extraction form where necessary. Any modifications arising from this pilot phase will be documented and justified in the final scoping review.

The following minimum data will be extracted:

- Bibliographic information: author(s), year of publication, country, and publication source.
- Population: characteristics of participating students and/or teachers, year or level of study, and sample size.
- Concept: didactic strategies implemented; classification of the authentic engineering problem according to the four operational criteria defined above: (A) mathematical centrality, (B) engineering situatedness, (C) empirical grounding of data, and (D) functional use of the derivative; evidence supporting the assessment of each criterion; overall classification as an authentic engineering problem based on the simultaneous fulfillment of criteria A–D; type of authentic engineering problem; and digital technologies used, when applicable.
- Context: country, institution, and course in which the educational experience was implemented (e.g., differential calculus, applied mathematics).
- Study methods: study design, methodological approach (qualitative, quantitative, or mixed methods), and data collection instruments.
- Key findings: reported learning difficulties, main conclusions, and relevant didactic recommendations.

A draft of the data extraction form is presented in Annex 2. The form may be refined during the review process as necessary to accommodate the characteristics of the included studies. Any modifications will be documented and justified in the final review.

Where reported information is incomplete or unclear, the review team may contact the corresponding authors to request clarification or additional information. This procedure aims to ensure the completeness, consistency, and accuracy of the extracted data.

In accordance with the methodological guidance provided by the JBI Manual for Evidence Synthesis [32], no formal critical appraisal of methodological quality will be undertaken, as the purpose of a scoping review is to map the extent, nature, and characteristics of the available evidence rather than to evaluate the methodological quality of the included studies. Accordingly, data extraction will focus on describing the characteristics of the included studies and identifying instructional approaches, educational contexts, and principal findings reported in the literature.

### 2.6 Analysis and synthesis

The evidence will be organized to address directly the objective and research question of this scoping review. Consistent with the descriptive and exploratory nature of scoping reviews, the findings will be synthesized using complementary narrative, tabular, and visual approaches.

The analysis will be conducted at three levels

### Descriptive and thematic synthesis

The analysis will begin with a descriptive and thematic synthesis aimed at identifying patterns, trends, and research gaps across the included studies. Findings will be grouped into categories developed from the evidence reported in the primary studies through an inductive thematic content analysis approach, following the JBI Manual for Evidence Synthesis [32]. This approach will facilitate the identification of patterns, convergences, and divergences across the literature. In addition, descriptive statistics (frequencies and distributions) will be used to summarize the characteristics of the included studies and support the tabular and graphical presentation of the results.

### Visual and tabular synthesis

To complement the narrative synthesis, the findings will also be presented through visual and tabular formats to facilitate comparison and interpretation.

These will include:

- Summary tables organizing the extracted information (e.g., didactic strategies versus types of authentic engineering problems, or digital technologies versus reported learning difficulties).
- Conceptual maps or diagrams illustrating the relationships among the identified didactic strategies, educational contexts, and reported difficulties.
- Descriptive figures showing the distribution of the evidence by publication year, country, or type of digital technology used.

### Integrated narrative synthesis

Finally, an integrated narrative synthesis will bring together the findings from the previous stages and relate them to the objective and research question of the review. This synthesis will highlight implications for teaching practice in engineering education, identify research gaps, and suggest directions for future research.

Neither meta-analysis nor quantitative synthesis will be undertaken, as the purpose of this scoping review is to map the extent, nature, and characteristics of the available evidence rather than to estimate the effectiveness or impact of the instructional strategies.

### 2.7 Ethical considerations and registry

Since this scoping review is based exclusively on the analysis of publicly available secondary sources, it does not involve human participants or the collection of personal data and therefore does not require approval from a research ethics committee.

The protocol has been registered in the Open Science Framework (OSF) and assigned the identifier DOI 10.17605/OSF.IO/T4BEA. This registration promotes transparency, methodological traceability, and reproducibility, while allowing future updates should substantial modifications to the protocol become necessary. In addition, all materials generated during the review process, including the data extraction forms, PRISMA-ScR checklist, flow diagram, and extraction matrices, will be deposited in an institutional repository and made openly available upon publication of the review.

### 2.8 Study status and timeline

At the time of this revision, the review is at the protocol stage. Record screening has not yet begun, and data extraction has not been initiated. No study selection or pilot screening has been conducted.

We anticipate completing record screening by November 2026, data extraction by December 2026, and the synthesis and reporting of results by January 2027.

## 3. Chronogram, data management, and dissemination of results

### 3.1. Work timeline

The review will be conducted in six consecutive stages, from protocol development to dissemination of the findings, over an estimated period of eight months.

Stage 1. Protocol development, conceptual refinement, and registration (Months 1–3): review of methodological guidance, refinement of the research question, preparation of the protocol, and registration in OSF (https://doi.org/10.17605/OSF.IO/T4BEA).

Stage 2. Search strategy (Month 4): development and validation of the search strategies, execution of database searches, and complementary backward and forward snowballing.

Stage 3. Study selection (Months 4–5): title and abstract screening, full-text assessment in Rayyan, pilot assessment of the eligibility criteria, and resolution of reviewer disagreements through consensus.

Stage 4. Data extraction (Months 5–6): pilot testing of the data extraction form, extraction of data from the included studies, and consolidation of the database.

Stage 5. Analysis and synthesis (Month 7): preparation of evidence maps, summary tables, descriptive figures, and integrated narrative synthesis.

Stage 6. Manuscript preparation and dissemination (Month 8): preparation of the final manuscript, internal revision by the research team, and submission to an indexed journal.

The timeline may be adjusted according to the volume of identified studies or reviewer availability. Any substantial modifications to the protocol or timeline will be documented through updates to the OSF registration.

### 3.2. Data management and reproducibility

All bibliographic records and relevant documents will be stored in a secure institutional repository with a structured folder organization. References will be managed using Rayyan (QCRI), and the extracted data will be organized in Microsoft Excel. Working documents, including the data extraction forms, spreadsheets, PRISMA-ScR checklist, flow diagram, and coding framework, will be deposited in the Open Science Framework (OSF) repository associated with the project to ensure transparency, traceability, and reproducibility of the review process. Upon publication of the review, the complementary materials will be made openly available under the Creative Commons Attribution 4.0 International License (CC BY 4.0).

### 3.3. Dissemination plan

The findings of this scoping review will be disseminated through: (a) publication in a peer-reviewed open-access journal; (b) presentation at international conferences on engineering education and mathematics education; and (c) public availability of the review protocol, data extraction forms, and final dataset through the Open Science Framework (OSF) repository.

This dissemination strategy aims to contribute to strengthening the teaching and learning of calculus in engineering education and to support the adoption of evidence-informed teaching practices in higher education.

## Supporting information

S1 File Reproducible search strategy. Complete database-specific search strategies developed for the scoping review.
(DOCX)

S2 File PRISMA-ScR checklist. Revised PRISMA-ScR checklist for the scoping review protocol.
(DOCX)

S3 File Data extraction matrix. Data extraction form developed for the scoping review.
(DOCX)
