# Supplementary material for: Teaching and learning strategies for the derivative in engineering education: A scoping review protocol
Source: PLoS One. 2026 Oct 22;21(10):e0360253. doi: 10.1371/journal.pone.0360253 (PMC13690235; doi:10.1371/journal.pone.0360253)
Supplement: Supplementary file 1 [file pone.0360253.s001.docx]

# Annex 1. Reproducible Search Strategy (JBI standard template)

# This annex documents the reproducible search strategy used in this review, following the guidelines of the JBI Manual for Evidence Synthesis (2020) and the transparency criteria established by PRISMA-ScR. The databases consulted, the exact search strings, the search fields used, the dates, the filters applied, and the documentation required to ensure replicability are described.

## 1. Databases consulted

The systematic search will be conducted in the following databases, recognized for their coverage in mathematics education, engineering education, and educational technology:

- **Scopus**
- **Web of Science**
- **ERIC**
- **SciELO**

# These databases were selected because of their extensive coverage of peer-reviewed literature in mathematics education, engineering education, and educational technology. Scopus and Web of Science provide broad multidisciplinary coverage of indexed journals, ERIC specializes in educational research, and SciELO offers relevant visibility of scientific production from Latin America and Iberian contexts.

# 2. Reproducible search strategies

The search strategy was designed using the elements of the PCC framework established in the protocol:

- **Population:** undergraduate students enrolled in engineering programs.
- **Concept:** didactic strategies related to the derivative that integrate authentic problems and/or digital technologies.
- **Context:** higher education engineering programs.

Boolean operators, truncation symbols, quotation marks for exact terms, and syntax adapted to each database were used.

The search strategy was iteratively refined by the research team to improve the relevance and sensitivity of the search strings. Adjustments were made to the keywords and Boolean combinations to balance comprehensiveness and specificity. The final search strings were adapted to the syntax and indexing systems of each database.

# 2.1 Search strategy – Web of Science (WoS)

**Collection:** Web of Science Core Collection

**Search field:** Topic (TS)

**Filters**

- Years: 2000–2026
- Peer-reviewed publications
- Languages: English, Spanish, Portuguese
- Document type: Articles and book chapters

**WoS search string**

TS = (("engineering student*" OR "engineering education" OR "undergraduate engineering" OR "engineering program*") AND (derivat* OR "differential calculus" OR "rate of change" OR "tangent line" OR "optimization problem*" OR "calculus education" OR "teaching strateg*" OR "didactic strateg*" OR "instructional strateg*" OR "teaching method*" OR "instructional design" OR "pedagogical approach*" OR "teaching practice*" OR "learning activit*" OR "learning task*" OR "authentic problem*" OR "real-world problem*" OR "real-life problem*" OR "authentic task*" OR "applied problem*" OR "contextualized problem*" OR "engineering context*" OR "simulation context*") AND ("higher education" OR universit* OR college* OR undergraduate* OR postsecondary)).

# 2.2 Search strategy – Scopus

**Search field:** TITLE-ABS-KEY

**Filters**

- 2000–2026
- Peer-reviewed articles and book chapters
- Languages: English, Spanish, Portuguese

**Scopus search string**

TITLE-ABS-KEY (("engineering student*" OR "engineering education" OR "undergraduate engineering" OR "engineering program*") AND (derivat* OR "differential calculus" OR "rate of change" OR "tangent line" OR "optimization problem*" OR "calculus education" OR "teaching strateg*" OR "didactic strateg*" OR "instructional strateg*" OR "teaching method*" OR "instructional design" OR "pedagogical approach*" OR "teaching practice*" OR "learning activit*" OR "learning task*" OR "authentic problem*" OR "real-world problem*" OR "real-life problem*" OR "authentic task*" OR "applied problem*" OR "contextualized problem*" OR "engineering context*" OR "simulation context*") AND ("higher education" OR universit* OR college* OR undergraduate* OR postsecondary)).

**2.3 Search strategy – ERIC**

**Search field:** Title, Abstract, Descriptors

**Filters**

- Peer-reviewed
- 2000–2026
- Languages: English, Spanish, Portuguese

**ERIC search string**

# (("engineering student*" OR "engineering education" OR "undergraduate engineering" OR "engineering program*") AND (derivat* OR "differential calculus" OR "rate of change" OR "tangent line" OR "optimization problem*" OR "calculus education" OR "teaching strateg*" OR "didactic strateg*" OR "instructional strateg*" OR "teaching method*" OR "instructional design" OR "pedagogical approach*" OR "teaching practice*" OR "learning activit*" OR "learning task*" OR "authentic problem*" OR "real-world problem*" OR "real-life problem*" OR "authentic task*" OR "applied problem*" OR "contextualized problem*" OR "engineering context*" OR "simulation context*") AND ("higher education" OR universit* OR college* OR undergraduate* OR postsecondary)).

# 2.4 Search strategy – SciELO

**Fields used:** Title, Abstract, Subject

**Filters**

- Peer-reviewed articles
- 2000–2026
- Languages: English, Spanish, Portuguese

**SciELO search string**

# (("engineering student*" OR "engineering education" OR "undergraduate engineering" OR "engineering program*") AND (derivat* OR "differential calculus" OR "rate of change" OR "tangent line" OR "optimization problem*" OR "calculus education" OR "teaching strateg*" OR "didactic strateg*" OR "instructional strateg*" OR "teaching method*" OR "instructional design" OR "pedagogical approach*" OR "teaching practice*" OR "learning activit*" OR "learning task*" OR "authentic problem*" OR "real-world problem*" OR "real-life problem*" OR "authentic task*" OR "applied problem*" OR "contextualized problem*" OR "engineering context*" OR "simulation context*") AND ("higher education" OR universit* OR college* OR undergraduate* OR postsecondary)).

# 3. Documentation

Each search will be documented including the database name, search date, exact search string used, filters applied, and the number of records retrieved. This documentation will ensure transparency and full reproducibility of the review process.

## All search records will be retained to allow verification and replication of the search process.
