# Supplementary material for: Teaching and learning strategies for the derivative in engineering education: A scoping review protocol
Source: PLoS One. 2026 Oct 22;21(10):e0360253. doi: 10.1371/journal.pone.0360253 (PMC13690235; doi:10.1371/journal.pone.0360253)
Supplement: Supplementary file 2 [file pone.0360253.s002.docx]

**PRISMA-ScR Compliance Checklist**

This checklist summarizes the extent to which the present protocol addresses the applicable reporting items of the PRISMA Extension for Scoping Reviews (PRISMA-ScR). Items applicable only to completed scoping reviews are indicated as “N/A”, as this manuscript describes a protocol. The corresponding location within the manuscript is provided for each applicable item.

| **Section** | **Item** | **PRISMA-ScR item** | **Reported?** | **Page(s)** |
| --- | --- | --- | --- | --- |
| TITLE | 1 | Title | Included | p.1 |
| ABSTRACT | 2 | Structured summary | Included | p.1–2 |
| INTRODUCTION | 3 | Rationale | Included | p.2–4 |
|  | 4 | Objectives | Included | p.4 |
| METHODS | 5 | Protocol and registration | Included | p.11 |
|  | 6 | Eligibility criteria | Included | p.5–7 |
|  | 7 | Information sources | Included | p.7–8 |
|  | 8 | Search | Included | p.7–9 |
|  | 9 | Selection of sources of evidence | Included | p.8–9 |
|  | 10 | Data charting process | Included | p.9–10 |
|  | 11 | Data items | Included | p.9–10 |
|  | 12 | Critical appraisal of individual sources of evidence (optional) | Included | p.10 |
|  | 13* | Synthesis of results | Included | p.10–11 |
| RESULTS | 14 | Selection of sources of evidence | N/A | N/A |
|  | 15 | Characteristics of sources of evidence | N/A | N/A |
|  | 16 | Critical appraisal within sources of evidence (optional) | N/A | N/A |
|  | 17 | Results of individual sources of evidence | N/A | N/A |
|  | 18 | Synthesis of results | N/A | N/A |
| DISCUSSION | 19 | Summary of evidence | N/A | N/A |
|  | 20 | Limitations | N/A | N/A |
|  | 21 | Conclusions | N/A | N/A |
| FUNDING | 22 | Funding | Included | p.13 |

* For protocol manuscripts, Item 13 refers to the planned approach for data synthesis described in the Methods section.

**Reference**

Tricco AC, Lillie E, Zarin W, O’Brien KK, Colquhoun H, Levac D, Peters MDJ, et al. PRISMA extension for scoping reviews (PRISMA-ScR): Checklist and explanation. Ann Intern Med. 2018;169(7):467–473. doi:10.7326/M18-0850.
