# Supplementary material for: Teaching and learning strategies for the derivative in engineering education: A scoping review protocol
Source: PLoS One. 2026 Oct 22;21(10):e0360253. doi: 10.1371/journal.pone.0360253 (PMC13690235; doi:10.1371/journal.pone.0360253)
Supplement: Supplementary file 3 [file pone.0360253.s003.docx]

**Annex 2. Data extraction matrix (Preliminary form)**

The following matrix will serve as the preliminary data extraction form to be used by reviewers during the review process. This instrument may be iteratively refined according to the nature of the studies included, following the methodological recommendations of the JBI Manual for Evidence Synthesis.

| **Section** | **Variable to be extracted** | **Description / Instructions** |
| --- | --- | --- |
| **1. Study identification** | Study ID | Internal code assigned by the research team. |
|  | Author(s) | Record the authors as they appear in the publication. |
|  | Year of publication | Year indicated in the source. |
|  | Study title | Full title of the publication. |
|  | Country | Country where the study was conducted. |
|  | Source / Journal | Name of the journal, conference proceedings, or publication source. |
|  | Document type | Empirical study; didactic experience; intervention proposal; theoretical study; review study; other (specify). |
| **2. Population**  **(PCC – P)** | Student characteristics | Engineering education level, engineering program, and participant characteristics. |
|  | Sample size | Number of participants and selection criteria. |
| **3. Concept**  **(PCC – C)** | Didactic strategies | Description of the didactic strategy implemented. |
|  | Criterion A – Mathematical centrality | Record whether the derivative is mathematically necessary to solve or interpret the task (Yes/No) and provide supporting evidence from the study. |
|  | Criterion B – Engineering situatedness | Record whether the task is situated in a recognizable engineering context involving relevant variables, constraints, or decisions (Yes/No) and provide supporting evidence from the study. |
|  | Criterion C – Empirical grounding of data | Record whether the task uses realistic, measured, simulated, or disciplinary data or parameters (Yes/No) and provide supporting evidence from the study. |
|  | Criterion D – Functional use of the derivative | Record whether the derivative is used for an engineering purpose, such as prediction, optimization, control, design, or interpretation of system behavior (Yes/No) and provide supporting evidence from the study. |
|  | Overall authenticity classification | Classify the task as an authentic engineering problem only when criteria A–D are simultaneously satisfied (Yes/No). |
|  | Type of authentic engineering problem | Describe the type of authentic engineering problem reported (e.g., optimization, rates of change, prediction, control, design, or interpretation of system behavior). |
|  | Resources / digital technologies | Record the digital technologies used, when applicable (e.g., GeoGebra, MATLAB, interactive simulations, digital platforms). |
|  | Mathematical content | Derivative, differential calculus concepts, rates of change, optimization, etc. |
| **4. Context (PCC)** | Educational context | Higher education setting, engineering discipline, and course level. |
|  | Institution / Program | Institution, country, engineering program, and course. |
|  | Instructional modality | Face-to-face, hybrid, or online. |
|  | Instructor characteristics (when reported) | Profile, role, and number of instructors involved in the instructional strategy (when reported). |
| **5. Study methods** | Methodological approach | Qualitative, quantitative, or mixed methods. |
|  | Research design | Case study, quasi-experimental study, etc. |
|  | Instruments | Observation protocols, questionnaires, interviews, rubrics, tests, etc. |
|  | Duration | Weeks or semesters of implementation. |
| **6. Results and findings** | Main findings | Main conclusions reported in the study. |
|  | Reported difficulties | Reported learning difficulties related to the derivative and its application. |
|  | Recommendations | Relevant didactic recommendations reported in the study. |
| **7. Relevance for the review** | Alignment with the objective | How the study contributes to the evidence mapping. |
|  | Notable aspects | Innovative or distinctive elements identified. |
| **8. Additional information** | Reviewer notes | Observations or inconsistencies detected during extraction. |
|  | Supplementary files or links | Supplementary materials associated with the study. |
